# Indoor wet cells as a habitat for melanized fungi, opportunistic pathogens on humans and other vertebrates

**DOI:** 10.1038/s41598-018-26071-7

**Published:** 2018-05-16

**Authors:** Xiaofang Wang, Wenying Cai, A. H. G. Gerrits van den Ende, Junmin Zhang, Ting Xie, Liyan Xi, Xiqing Li, Jiufeng Sun, Sybren de Hoog

**Affiliations:** 1 Department of Dermatology, Sun Yat-sen Memorial Hospital, Sun Yat-sen University, Guangzhou, China; 2 Department of Dermatology and Venerology, Guangming New District Central Hospital, Shenzhen, Guangdong Province China; 3 Westerdijk Fungal Biodiversity Institute, Utrecht, The Netherlands; 4 Department of Dermatology, The Second Affiliated Hospital, Guangzhou University of Chinese Medicine, Guangzhou, China; 5 Dematology Hospital of Southern Medical University, Guangzhou, China; 6 Guangdong Provincial Institute of Public Health, Guangdong Provincial Center for Disease Control and Prevention, Guangzhou, Guangdong China; 7 Center of Expertise in Mycology of Radboudumc/Canisius Wilhelmina Hospital, Nijmegen, The Netherlands; 8 Basic Pathology Department, Federal University of Paraná State, Curitiba, Paraná Brazil; 9 Department of Dermatology, First Hospital of Peking University, Beijing, China

## Abstract

Indoor wet cells serve as an environmental reservoir for a wide diversity of melanized fungi. A total of 313 melanized fungi were isolated at five locations in Guangzhou, China. Internal transcribed spacer (rDNA ITS) sequencing showed a preponderance of 27 species belonging to 10 genera; 64.22% (n = 201) were known as human opportunists in the orders *Chaetothyriales* and *Venturiales*, potentially causing cutaneous and sometimes deep infections. *Knufia epidermidis* was the most frequently encountered species in bathrooms (n = 26), while in kitchens *Ochroconis musae* (n = 14), *Phialophora oxyspora* (n = 12) and *P. europaea* (n = 10) were prevalent. Since the majority of species isolated are common agents of cutaneous infections and are rarely encountered in the natural environment, it is hypothesized that indoor facilities explain the previously enigmatic sources of infection by these organisms.

## Introduction

Black yeast-like and other melanized fungi are frequently isolated from clinical specimens and are known as etiologic agents of a gamut of opportunistic infections, but for many species their natural habitat is unknown and hence the source and route of transmission remain enigmatic. The majority of clinically relevant black yeast-like fungi belong to the order *Chaetothyriales*, while some belong to the *Venturiales*. Propagules are mostly hydrophilic^1^ and reluctantly dispersed by air, infections mostly being of traumatic origin. Members of the group are associated with mild implantation diseases of skin and nails or chronic mutilating infections such as chromoblastomycosis and phaeohyphomycosis, but some show neurotropic dissemination. All infections may occur in immunocompetent individuals. Since special methods are required for their isolation from nature, a low competitive ability towards fast-growing contaminants has been hypothesized^2^.

In the environment, black yeast-like fungi have been recovered from unexpected, rather hostile (micro)environments, including rock, creosote-treated wood, hydrocarbon-polluted soil, and lichens^3–5^. Recent studies have demonstrated that black yeast-like fungi of *Chaetothyriales*, particularly *Exophiala* and *Cladophialophora* species are common colonizers in indoor wet cells^6,7^. The thermotolerant species *Exophiala dermatitidis* is frequent in steambaths, house baths and dishwashers^8,9^. Southeast Asia countries have a similar (sub)tropical climate, hot moisture sustaining thermophilic fungal growth during a large part of the year, although with marked geographical differences. However, disseminated *Exophiala dermatitidis* infections are particularly observed in East Asia countries^10^, e.g. Thailand, while the neurotropic species *Cladophialophora bantiana* occurs relatively often in India^11^. In order to understand the origin of infections, screening of households for fungi other than the common airborne fraction mainly involved in allergy (*Aspergillus, Alternaria, Cladosporium*) seems overdue. Seveal selective techniques have been developed enabling recovery the black yeast-like fungi^2,7^. In the present study we focused on black fungi in Sourthern China, where chromoblastomycosis and phaeohyphomycosis are endemic^12–16^. Our aim was to identify the spectum of black yeast-like fungi in indoor wet cells, and clarify whether these facilities harbour potential etiologic agents of disease.

## Methods

### Sampling and isolation

Environmental samples were collected from 53 families living in five regions (Yue Xiu, Li Wan, Bai Yun, Tian He, Hai Zhu) of Guangzhou city, during July to September, 2015. Black biofilms were sampled from bathrooms (water ladle, wall, soap box, washbasin, door, sprinklers, toothbrush cup, floor, brush, mirror), kitchen (water tank, chopping board, sterilizing cabinet, wall, cooking bench), refrigerator (rubber seal), washing machine (rubber seal, water tap) and water dispenser (water tank) using sterile cotton swabs and immediately contained in sterile tubes. The sampling tubes were stored for max. 24 h at room temperature before processing.

**Figure 1:**
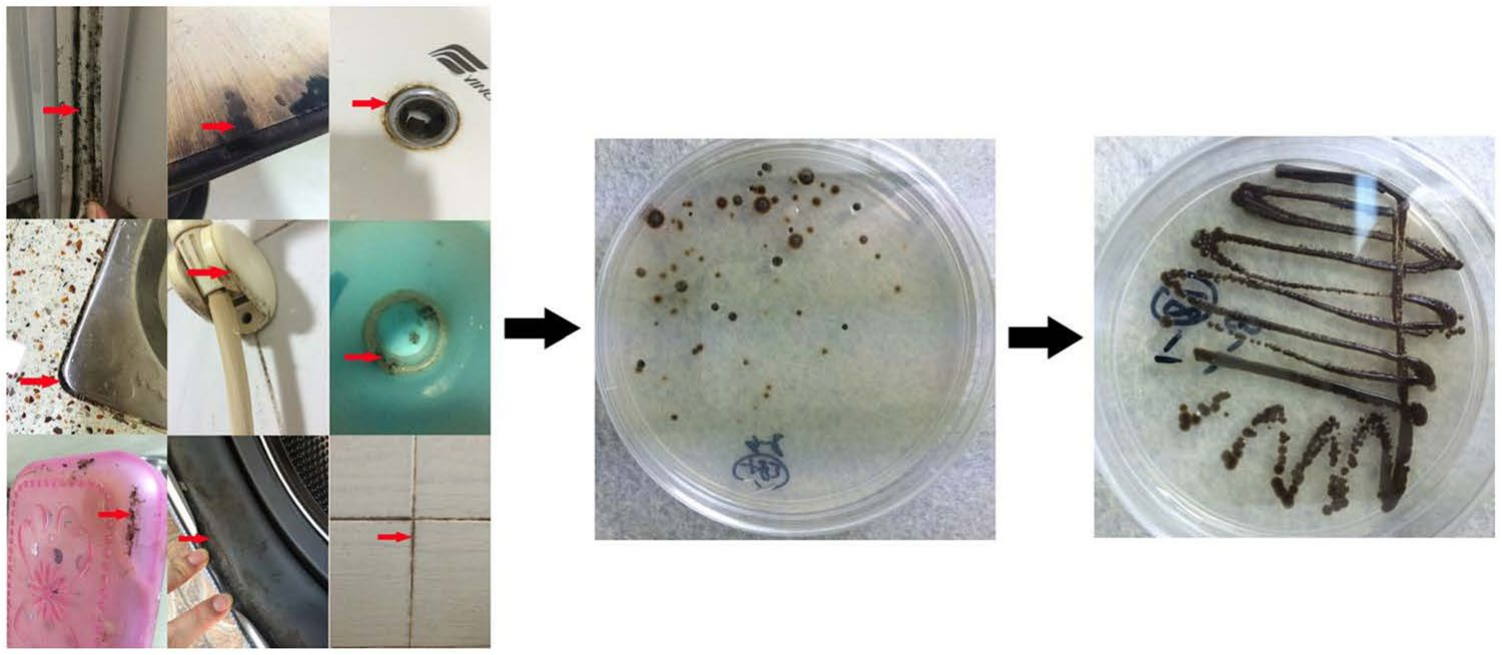
The workflow of sampling and isolation. The black biofilms samples from particular areas of bathroom, kitchen, refrigerator rubber seal, and washing machine were taken by using sterile cotton swabs; the black biofilm materials were cultured on PDA medium (200 mg/L chloramphenicol and 200 mg/L cycloheximide) for 2 weeks. Susceptive black yeast like single colony strains were taken for further culture under same condition as above.

For the inoculation and culture of suspect fungus, 500 gram of Potato Dextrose Agar (PDA) (Oxoid, Thermo Fisher Scientific, Basingstoke, UK) powder was solved in 1.28 liter demi water, and autoclaved under 121 °C, 105 kPa for 30 min. Each 250 mg of Chloramphenicol (Sigma Aldrich, St. Louis, MO.USA) and cycloheximide (Sigma Aldrich, St. Louis, MO.USA) were added into the PDA medium when it reach to about 60 °C, and mixted sufficiently before plating. Sterile cotton swabs contained the suspect fungus specimens were rubbed over the surface of PDA medium plate with 200 mg/L chloramphenicol and 200 mg/L cycloheximide, and incubated at 28 °C for 2 weeks. The workflow of sampling and isolation are shown in Fig. 1.

### Morphology

After 2 weeks culturing, the suspected slow-growing, blackish-brown colonies in the same plate were viewed and compared. The predominant colony type in each plate was considered as target colony, and one was selected and transferred to new growth media on PDA using a loop for further culture. Preliminary identification at the generic level was carried out by colony appearance and morphology by microscopy using slide cultures on Sabouraud’s glucose agar (SDA) (Oxoid, Thermo Fisher Scientific, Basingstoke, UK) after 2 weeks culturing at room temperature.

### DNA extraction

Methods of DNA extraction were those of Sun *et al*.^13^. Briefly, about 1 cm^2^ mycelium or yeasts of 15-d-old cultures was transferred to a 2 mL Eppendorf tube containing 300 μL CTAB (cetyltrimethylammonium bromide) buffer [CTAB 2% (w/v), NaCl 1.4 M, Tris-HCl 100 mM, pH 8.0; EDTA 20 mM, b-mercaptoethanol 0.2% (v/v)] and about 80 mg of a silica mixture (silica gel H, Merck 7736, Darmstadt, Germany/Kieselguhr Celite 545, Machery, Düren, Germany, 2:1, w/w). Cells were disrupted with sterile glass beads (Sigma, St. Louis, MO, USA) for approximately 5 min. Subsequently 200 μL CTAB buffer was added, the mixture was vortexed and incubated for 10 min at 65 °C. After addition of 500 μL chloroform, the solution was mixed and centrifuged for 5 min at 13, 000 rpm and the supernatant transferred to a new tube with 2 vols of ice cold 96% ethanol. DNA was allowed to precipitate for 30 min at −20 °C and then centrifuged again for 5 min at 13, 000 rpm. Subsequently the pellet was washed with cold 70% ethanol. After drying at room temperature it was resuspended in 97.5 μL TE-buffer plus 2.5 μL RNAse 20 U.mL^−1^ and incubated for 5 min at 37 °C, before storage at −20 °C.

### Sequencing and molecular identification

Ribosomal DNA (rDNA, codes for ribosomal RNA) Internal Transcribed Spacer (ITS) was amplified using primers V9G and LS266 and sequenced with ITS1 and ITS4^2^. Amplicons were cleaned with GFX PCR DNA purification kit (GE Healthcare, U.K.). Sequencing was performed on an ABI 3730XL automatic sequencer. Sequences were edited using the Seqman package (DNAStar, Madison, U.S.A.) and aligned using BioNumerics (Applied Maths, Sint-Martens-Latem, Belgium). Sequences were compared in a research data database of black fungi maintained at Westerdijk Fungal Biodiversity Institute, Utrecht, The Netherlands, and validated by ex-type strains of known species. In addition, all the ITS sequences were deposited to GenBank database, and conducted Blastn analysis (https://blast.ncbi.nlm.nih.gov/Blast.cgi) as default parameters settings. The most hits sequence of known species was considered as the target species showed in Supplementary (Table S1).

### Statistical analysis

Descriptive statistics were carried out with the statistical software package Statistical Product and Service Solutions (SPSS, 17.0) (IBM SPSS, Chicago, USA). Mann-Whitney U test for unparametric variables was used for comparison of strains among five different regions. The strains isolated in each family and location were also analyzed using one way ANOVA parametric test, followed with Turkey analysis. For all statistical tests, *p*-value of <0.05 was considered statistically significant.

## Results

A total of 509 samples were taken from 53 families at five regions of Guangzhou, China, of which 399 samples showed positive growth of melanized fungi (Table 1). Subsequently, 313 (61.49%) strains of black yeast-like fungi were isolated from 399 positive samples (Table 2). Eighty-six suspected colonies could not successfully be isolated during subsequent subculturing. Although the percentages of positive isolation per region ranged from 59.48% to 71.88%, no significant differences of total isolates were found between five regions investigated either based on unparametric Mann-Whitney U test (*p* = 0.427) or one way ANOVA parametric test *(p* = 0.671), followed by Turkey analysis (*p* = 0.714–0.999) (Table 2).

**Table 1:** Summary of samples and strains in different regions of Guangzhou, China.

	Yue Xiu	Li Wan	Bai Yun	Tian He	Hai Zhu	In total
Total samples (%)^a^	45 (8.84%)	93 (18.28%)	33 (6.49%)	32 (6.29%)	306 (60.12%)	509
Positive samples (%)^b^	43 (10.78%)	86 (21.56%)	28 (7.02%)	26 (6.52%)	216 (54.14%)	399
Strains (%)^c^	32 (10.23%)	56 (17.90%)	20 (6.39%)	23 (7.35%)	182 (58.15%)	313
Positive isolation percentage (%)^d^						61.50%

^a^percentage of samples in each regions versus total samples.

^b^percentage of melanized fungi culturing positive samples in each regions versus total positive samples.

^c^percentage of identified strains in each regions versus total strians.

^d^percentage of total strains versus total samples in each regions.

**Table 2:** Strains isolated in different regions of Guangzhou, China in this study (n = 313).

Families in each region	Haizhu	Liwan	Baiyun	Yuexiu	Tianhe	In total
1	15	0	0	0	0	15
2	8	0	0	0	0	8
3	9	0	0	0	0	9
4	2	0	0	0	0	2
5	4	0	0	0	0	4
6	4	0	0	0	0	4
7	6	0	0	0	0	6
8	5	0	0	0	0	5
9	6	0	0	0	0	6
10	5	0	0	0	0	5
11	20	0	0	0	0	20
12	9	0	0	0	0	9
13	5	0	0	0	0	5
14	6	0	0	0	0	6
15	7	0	0	0	0	7
16	2	0	0	0	0	2
17	3	0	0	0	0	3
18	3	0	0	0	0	3
19	5	0	0	0	0	5
20	7	0	0	0	0	7
21	6	0	0	0	0	6
22	4	0	0	0	0	4
23	5	0	0	0	0	5
24	8	0	0	0	0	8
25	7	0	0	0	0	7
26	2	0	0	0	0	2
27	5	0	0	0	0	5
28	2	0	0	0	0	2
29	3	0	0	0	0	3
30	9	0	0	0	0	9
31	0	12	0	0	0	12
32	0	3	0	0	0	3
33	0	7	0	0	0	7
34	0	9	0	0	0	9
35	0	6	0	0	0	6
36	0	5	0	0	0	5
37	0	4	0	0	0	4
38	0	3	0	0	0	3
39	0	7	0	0	0	7
40	0	0	9	0	0	9
41	0	0	4	0	0	4
42	0	0	7	0	0	7
43	0	0	0	3	0	3
44	0	0	0	8	0	8
45	0	0	0	0	0	0
46	0	0	0	12	0	12
47	0	0	0	9	0	9
48	0	0	0	0	3	3
49	0	0	0	0	2	2
50	0	0	0	0	5	5
51	0	0	0	0	2	2
52	0	0	0	0	7	7
53	0	0	0	0	4	4
Strains (%)^a^	182 (58.15%)	56 (17.90%)	20 (6.39%)	32 (10.23%)	23 (7.35%)	313

^a^Percentage of identified strains in each region *versus* total number of strains.

Initial identification of suspected isolates based on colony appearance and slide culture micromorphology was approximate. Most isolates were identified at genus level due to similar colony and morphology appearance under microscopy, e.g. the genera *Exophiala*, *Cladophialaphora*, *Phialophora*, as well as *Ochroconis* in the order *Venturiales*. Therefore, the molecular based identification became essential for classification of extremely similar species in morphology. The rDNA ITS sequences of all isolates were amplified and sequenced successfully. Local blast in a research database on black yeast-like fungi at Westerdijk Fungal Biodiversity Institute, identified 259 strains with 27 species distributed in 10 genera, including *Exophiala* (n = 77), *Phialophora* (n = 44), *Ochroconis* (n = 38), *Knufia* (n = 37), *Cladosporium* (n = 37), *Rhinocladiella* (n = 7), *Cyphellophora* (n = 7), *Hortaea* (n = 7), *Cladophialophora* (n = 4), and *Veronaea* (n = 1) (Table 3). The most frequently isolated species were *K. epidermidis* (n = 37, 11.82%), *O. musae* (n = 32, 10.22%), *P. oxyspora* (n = 27, 7.99%), *Cladosporium halotolerans* (n = 21, 6.93%), *E. lecanii-corni* (n = 18, 5.94%), *P. europaea* (n = 18, 5.75%), *E. alcalophila* (n = 13, 4.15%), *Cladosporium irritans* (n = 13, 4.15%) and *E. oligosperma* (n = 10, 3.19%). Apparently undescribed species were detected in the genera *Exophiala*, *Cyphellophora*, *Ochroconis*, *Hortaea* and *Cladophialophora* (Table 3). Fifty-four isolates were identified as unknown species based on either morphology or on rDNA ITS sequences (17.25%) (Table 3).

**Table 3:** Species identification of the black yeast-like fungi (n = 313).

Order	Genus	Species	Isolates (n)	Positive (%)
*Chaetothyriales* (n = 177)	*Exophiala* (n = 77)	*Exophiala lecanii-corni*	18	5.94%
		*Exophiala alcalophila*	13	4.15%
		*Exophiala oligosperma*	10	3.19%
		*Exophiala cancerae*	9	2.88%
		*Exophiala equina*	9	2.88%
		*Exophiala* sp. (11% to *E. equina*)	1	0.32%
		*Exophiala dermatitidis*	5	1.60%
		*Exophiala mesophila*	3	0.96%
		*Exophiala* sp. (5% to *E. nishimurae*)	3	0.93%
		*Exophiala* sp.	2	0.64%
		*Exophiala xenobiotica*	2	0.64%
		*Exophiala* sp. (10% to *E. aquamarina*)	1	0.32%
		*Exophiala* sp. (5% to *E. jeanselmei*)	1	0.32%
	*Knufia* (n = 37)	*Knufia epidermidis*	37	11.82%
	*Phialophora* (n = 44)	*Phialophora oxyspora*	25	7.99%
		*Phialophora europaea*	18	5.75%
		*Phialophora verrucosa*	1	0.32%
	*Cyphellophora* (n = 7)	*Cyphellophora fusarioides* (1% difference)	5	1.60%
		*Cyphellophora* sp. (4% to *C. pluriseptata*)	2	0.64%
	*Rhinocladiella* (n = 7)	*Rhinocladiella similis*	7	2.24%
	*Cladophialophora* (n = 4)	*Cladophialophora boppii* (0.8% difference)	2	0.64%
		*Cladophialophora* sp. (2% to *C. boppii*)	1	0.32%
		*Cladophialophora immunda*	1	0.32%
	*Veronaea* (n = 1)	*Veronaea japonica* (6% difference)	1	0.32%
*Venturiales* (n = 38)	*Ochroconis* (n = 38)	*Ochroconis musae*	32	10.22%
		*Ochroconis humicola* (0.2% difference)	6	1.92%
*Capnodiales* (n = 37)	*Cladosporium* (n = 37)	*Cladosporium halotolerans*	21	6.93%
		*Cladosporium irritans* (0.7% difference)	13	4.15%
		*Cladosporium oxysporum/tenuissimum*	3	0.96%
*Dothideales* (n = 7)	*Hortaea* (n = 7)	*Hortaea werneckii*	5	1.60%
		*Hortaea* sp. (8% to *H. werneckii*)	2	0.64%
unclassified (n = 54)	unclassified (n = 54)	unknown	54	17.25%
Total	10	27	313	100%

To assist species identification of all 313 strains, the rDNA ITS sequences were compared by Blast analysis against archived sequences in GenBank, which is a widely used approach for fungal identification. Unfortunately, only 14/27 species were corrected identified compared to the quality-controlled database at Westerdijk Fungal Biodiversity Institute (Table S1). Six species were misidentified including *Cladosporium irritans* (0.7% difference) as *Toxicocladosporium banksiae*, *Cyphellophora fusarioides* (1% difference) as *Cyphellophora laciniata*, *E. cancerae* as *E. salmonis, O. humicola* (0.2% difference) as *O. mirabilis*, *O. musae* as *O. humicola* and *P. verrucosa* as *P. americana*. Eight species (*H. werneckii* with 8% difference included) were identified at the genus or order level, including *C. oxysporum/tenuissimum* as *Cladosporium* sp., *E. equina* (11% difference) as *Chaetothyriales sp*., *V. japonica* (6% difference) as *Chaetothyriales* sp., *H. werneckii* (8% difference) as *Acremonium* sp., as well as *E. equina*, *E. aquamarina* (10% difference), *E. nishimurae* (5% difference), *E. jeanselmei* (5% difference) as *Exophiala* sp. The remaining *Exophiala* sp. was misidentified as *E. alcalophila*. Interestingly, 44/54 isolates previously identified as unknown species hit 4 known species on GenBank with variable coverage (66–99%) and identity (86–98%), although with different morphology. Four of fifty-four isolates identified as *Chaetothyriales* sp. with coverage (95–99%) and identity (90–93%). The remaining 6/54 isolates were identified as *Herpotrichiellaceae* sp. (n = 3), *Exophiala* sp. (n = 2) and *Phialaphora* sp. (n = 1), respectively.

An association analysis between isolation frequency and sampling areas indicated that the bathroom (n = 147, 46.96%) was the most common isolation site, followed by kitchen (n = 116, 37.06%), refrigerator (n = 32, 10.22%), washing machine (n = 17, 5.43%) and water dispenser (n = 1, 0.32%) (Table 4). The distribution of isolates was significantly variable in each location, which is proven by either unparametric Mann-Whitney U test (*p* = 0.015) of total isolates between four locations (water dispensers were not included in this test due to small data point) or one way ANOVA parametric test *(p* = 0.033), but rejected by a successive Turkey analysis among four locations (*p* = 0.064–0.995).

**Table 4:** Strains distribution in each isolation location in this study (n = 313).

Families	Bathroom	Kitchen	Refrigerator	Washing machine	Water dispenser
1	7	4	1	3	0
2	7	1	0	0	0
3	5	1	0	3	0
4	1	0	0	1	0
5	2	0	1	1	0
6	1	2	1	0	0
7	1	3	2	0	0
8	4	0	1	0	0
9	2	4	0	0	0
10	5	0	0	0	0
11	11	6	0	3	0
12	6	3	0	0	0
13	3	1	0	1	0
14	1	5	0	0	0
15	4	3	0	0	0
16	0	2	0	0	0
17	0	3	0	0	0
18	0	3	0	0	0
19	3	2	0	0	0
20	3	1	3	0	0
21	3	2	1	0	0
22	2	2	0	0	0
23	1	4	0	0	0
24	8	0	0	0	0
25	4	3	0	0	0
26	2	0	0	0	0
27	2	1	2	0	0
28	1	1	0	0	0
29	1	0	1	1	0
30	8	1	0	0	0
31	5	4	3	0	0
32	2	1	0	0	0
33	4	3	0	0	0
34	4	4	1	0	0
35	0	5	1	0	0
36	1	2	2	0	0
37	3	1	0	0	0
38	2	1	0	0	0
39	1	3	1	2	0
40	5	4	0	0	0
41	3	1	0	0	0
42	3	4	0	0	0
43	1	2	0	0	0
44	3	5	0	0	0
45	0	0	0	0	0
46	3	6	2	0	1
47	1	1	7	0	0
48	1	1	0	1	0
49	1	0	1	0	0
50	2	3	0	0	0
51	1	1	0	0	0
52	2	4	0	1	0
53	1	2	1	0	0
Total	147 (46.96%)	116 (37.06%)	32 (10.22%)	17 (5.43%)	1 (0.32%)

The analysis between isolation frequency of each species and sampling areas showed that in bathrooms, the most frequently encountered species were *K. epidermidis* (n = 26)*, E. lecanii-corni* (n = 14), *P. oxyspora* (n = 12), *O. musae* (n = 12) and *E. alcalophila* (n = 10), while in kitchens, the most frequently isolated species were *O. musae* (n = 14)*, P. oxyspora* (n = 12), and *P. europaea* (n = 10) (Table 5). In refrigerators, *Cladosporium* species such as *C. halotolerans* were commonly encountered (n = 9). In washing machines and water dispensers, only few black fungal isolates were found other than *K. epidermidis* (n = 6) and *O. musae* (n = 5) (Table 5).

**Table 5:** Quantity and percentage of species in different sampled locations (n = 313).

Name	Total strain number	Kitchen	Bathroom	Refrigerator/rubber seal	Water dispenser	Washing machine/rubber seal
*Exophiala lecanii-corni*	18	4	14	0	0	0
*Exophiala alcalophila*	13	3	10	0	0	0
*Exophiala oligosperma*	10	8	0	2	0	0
*Exophiala cancerae*	9	1	7	1	0	0
*Exophiala equina*	9	3	6	0	0	0
*Exophiala equina* (11% difference)	1	1	0	0	0	0
*Exophiala dermatitidis*	5	4	0	1	0	0
*Exophiala mesophila*	3	1	2	0	0	0
*Exophiala nishimurae* (5% difference)	3	3	0	0	0	0
*Exophiala xenobiotica*	2	2	0	0	0	0
*Exophiala* species (0.3% difference)	2	0	0	1	0	1
*Exophiala jeanselmei* (5% difference)	1	1	0	0	0	0
*Exophiala aquamarina* (10% difference)	1	0	1	0	0	0
*Knufia epidermidis*	37	8	26	0	0	3
*Phialophora oxyspora*	25	12	12	1	0	0
*Phialophora europaea*	18	10	5	2	0	1
*Phialophora verrucosa*	1	1	0	0	0	0
*Cyphellophora fusarioides* (1% difference)	5	0	4	0	0	1
*Cyphellophora pluriseptata* (4% difference)	2	1	1	0	0	0
*Rhinocladiella similis*	7	5	1	1	0	0
*Cladophialophora boppii* (0.8% difference)	2	2	0	0	0	0
*Cladophialophora boppii* (2% difference)	1	0	1	0	0	0
*Cladophialophora immunda*	1	1	0	0	0	0
*Veronaea japonica* (6% difference)	1	1	0	0	0	0
*Ochroconis musae*	32	14	12	1	0	5
*Ochroconis humicola* (0.2% difference)	6	0	6	0	0	0
*Cladosporium irritans* (0.7% difference)	13	5	1	6	0	1
*Cladosporium halotolerans*	21	2	7	9	1	2
*Cladosporium oxysporum/tenuissimum*	3	0	1	1	0	1
*Hortaea werneckii*	5	2	1	2	0	0
*Hortaea werneckii* (8% difference)	2	1	0	0	0	1
Unknown species	54	20	29	4	0	1
Total	313	116	147	32	1	17
Percentage	100%	37.06%	46.96%	10.22%	0.32%	5.43%

## Discussion

Implantation of contaminated thorns or wooden splinters has been hypothesized to be a main infection route of chromoblastomycosis or phaeohyphomycosis, and therefore the agents of both diseases probably originate from the natural environment^2^. Systemic occurrence of black yeasts probably has a pulmonary route of infection, for which hot indoor wet cells have been suggested, such as steambaths^10^. We establish the prevalence of black yeast-like fungi in the wet cell environment, which thus far has been neglected in indoor studies. Most studies of indoor fungi focus on airborne fungi (*Alternaria*, *Cladosporium*, *Penicillium*, *Aspergillus*, *Rhizopus* and *Mucor*). Those fungi grow very fast compared with black yeast-like fungi, which make it difficult to recover black yeast by direct isolation. The fungal community described in this study is very different from that of studies focus on indoor airborne fungi. Only few studies focus on the black yeast and melanzied fungi in indoor wet cell environments are available. Clarification of the prevalence of melanized fungi in the wet cell environment is significant to estimate potential transmission routes of melanized fungi. In this study, 399 of 509 samples taken in moist indoor sampling sites of human residences in Guangzhou, Southern China proved to be positive for black yeast-like and other melanized fungi. The remaining 110 samples showed no growth of black yeast-like fungi or growth of airborne contaminent fungi, e.g. *Aspergillus* and *Penicillium* species. The black yeast genus *Exophiala*, containing species that reproduce by budding with slimy conidia, was common (n = 77), followed by *Knufia* (n = 37), and *Phialophora* (n = 44), while also many strains of *Rhinocladiella*, *Cyphellophora*, and *Cladophialophora* were encountered. All these genera belong to a single fungal order, *Chaetothyriales*. The genus *Ochroconis*, which is unrelated (order *Venturiales*) but has similar ecology as *Exophiala* was also common (n = 38). Except for *E. dermatitidis*, the majority of isolated fungi have been listed by de Hoog *et al*.^17^ as potential agents of mild skin and nail infections, confirming Lian and de Hoog^7^ who noticed that bathrooms harbour a remarkably large number of skin fungi that were until then not known from environmental sources. Lian and de Hoog^7^ hypothesized that human skin that is softened during bathing might be more vulnerable to fungal infection. Our study underlines that indoor wet areas serve as reservoir for fungi known to be involved in human infections^18^. Temperatures of our sampling sites usually do not exceed 37 °C, which might be a reason that only species were encountered that infect human skin and nails, rather than species causing systemic infections. In contrast, hot moist indoor environments such as steambaths^10^ and dishwashers^19^ are regularly colonized by thermophilic systemic opportunists, such as the black yeast *Exophiala dermatitidis* and the white yeasts *Candida parapsilosis* and *Magnusiomyces capitatus*.

*Exophiala* species are often isolated from indoor water sources, such as sinks, drainpipes, swimming pools, and bathing facilities, and also occur in municipal drinking water enabling biofilm formation^20,21^. Chaetothyrialean species of Table 3 are involved in mild cutaneous and nail mycoses^22^. Our isolation of *E. dermatitidis* from a kitchen (n = 4) and from the rubber seal of a refrigerator (n = 1) may be coincidental. The fungus can occasionally be found in sites with lower temperature, such as sinks and humidifiers, but is very abundant in steambaths^10^.

*Knufia epidermidis* was a common species in the bathroom. It was first described from a superficial skin lesion with blackish discoloration in an 80-yr-old Chinese patient^23^. Subsequent studies detected this species in human toes, skin and nails^22,24^.

*Phialophora europaea*, currently classified in *Cyphellophora*^25^, is among the fungi causing mild skin and nail infections^22,26,27^. The species thus far has rarely been found outside the human host, but we encountered it at high isolation rates in bathrooms and kitchens. The genus *Cyphellophora*, along with some species formerly classified in *Phialophora* phylogenetically separate from the main group of human opportunists in *Herpotrichiellaceae*. The two *Cyphellophora* species identified in this study suggest wide spread in indoor wet cells*. Cladophialophora boppii* seems to have a similar ecology, occasionally being found as a colonizer on human skin and nails^28^, as well as in bathrooms^22^. *Rhinocladiella similis* was originally described as the sympodial counterpart of *Exophiala jeanselmei*, and has occasionally been reported from human infections^29^. We isolated several strains from bathroom facilities, confirming data of Matos *et al*.^30^.

*Ochroconis musae* (order *Venturiales*) was the most frequently encountered species in kitchens and bathrooms, confirming findings of Lian and de Hoog^7^. The species was originally described as *O. mirabilis* from strains from humans and moist sources^31^, but Lu *et al*.^32^ had found a strain of the same species on a banana leaf and introduced the *O. musae* one month earlier. However, it is not a plant-associated fungus but is regularly associated with low-nutrient waters^31^. It occasionally causes skin and nail infections of immunocompetent patients^33,34^, and is particularly common as an invader of cold-blooded, waterborne vertebrates such as frogs and fish^31^. Yew *et al*.^35^ found that melanin production, osmoregulation, expanded gene family encoding taurine catabolism dioxygenase (*TauD*/*TdfA* domain), glutathione-S-transferase domains and RTA1-like protein families enabled the fungus to thrive under hostile oligotrophic conditions, such as low-nutrient and moist environments.

*Hortaea werneckii* (order *Capnodiales*) is a halophilic black yeast^36^. In humans it is known from superficial colonization the hand called tinea nigra^37,38^ which is explained by salty cutaneous conditions e.g. during beach holidays. Our consistent finding of the species in indoor environments demonstrates that the halotolerant amplitude of the fungus is very wide and includes oligotrophic habitats. The fungus has no opportunistic potential.

*Cladosporium* species (order *Capnodiales*) are airborne fungi, considered as biofilm formers and contaminants with no clinical significance. *Cladosporium halotolerans* was recently segregated from *C. sphaerospermum* as an osmotolerant fungus commonly isolated from hypersaline water of salterns and other environments with low water activity such as peanut shells^39^; it is regularly encountered on bathroom walls^40^. In humans *Cladosporium* species are isolated from the respiratory tract (54.5%), followed by superficial (28.4%) and deep tissues and fluids (14.7%)^41^, but very few unambiguously proven clinical cases exist. The most common isolation sites of *Cladosporium* species such as *C. irritans* were rubber seals of refrigerators and bathrooms, suggesting temperature-independent oligotrophism. *Cladosporium oxysporum/tenuissimum* was isolated from the same sites as *C. halotolerans*, indicating similar ecology. *Cladosporium irritans* otherwise has been reported from plant leaves and other environmental sources^42,43^ and from respiratory specimens without signs of invasion^41^. *Cladosporium* is preponderant in the airborne mycobiota and considered as important allergenic fungi^44^.

In conclusion, we noticed that most of the black fungi that were recovered from indoor wet cells mostly belong to the order *Chaetothyriales* and the family Herpotrichiellaceae within this order it otherwise known to contain numerous agents of superficial skin and nail infections. Maximum growth temperatures of most species are around 40 °C. Notably, the related fungus *Exophiala dermatitidis*, able to grow above 40 °C and found at higher temperatures in the indoor environment, viz. in steambaths and dishwashers, potentially causes disseminated, potential fatal human infections. *Exophiala* species with maximum growth temperatures around 33 °C are particularly found infecting waterborne cold-blooded animals^45^. This suggests that thermotolerance is an important virulence factor in black fungi, determining that the fungi which are not capable of growing under human conditions (33–37 °C) are not able to infect humans.

In addition to the above known black yeast-like fungi, a high rate of unknown strains (n = 54, 17.25%) was recovered in our dataset. The most interesting finding is that 44/54 isolates, which previously identified as unknown species against the black yeast database at Westerdijk Fungal Biodiversity Institute, hit 4 known species on GenBank. The remained 10 isolates were also identified in order or genus level. It suggests the stochastic blast analysis on a massive, multifarious nucleotides database, such as GenBank, may guide the further identification of novel species. However, when all 259 known strains sequences were blasted on this database, only 14/27 species hit the correct nucleotides sequences archived in GenBank. The remained 13 species were identified in genus level or completely misidentified. One reasonable explanation is that Genbank is a stock center for nucleotides sequences which deposited by researcher worldwide. The blast analysis only depend on the nucleotides sequences similarity, not refer to either morphology or other test. Another reason is that frequently nomenclature change of dispute species, thus this results suggest that well maintained and organized database will be the preference for novel isolates identification, e.g. the database at Westerdijk Fungal Biodiversity Institute for black yeast. Nevertheless, further studies are needed to describe these strains as novel taxa.

## Electronic supplementary material

Table S1
