# Supplementary material for: Indoor wet cells as a habitat for melanized fungi, opportunistic pathogens on humans and other vertebrates
Source: Sci Rep. 2018 May 16;8:7685. doi: 10.1038/s41598-018-26071-7 (PMC5955924; doi:10.1038/s41598-018-26071-7)
Supplement: Supplementary file 1 — Table S1 [file 41598_2018_26071_MOESM1_ESM.docx]

Supplementary Table 1. The detail information of 313 isolates in this study.

| Number | Isolates | GenBank accession number | Blast in black yeast database at Westerdijk Fungal Biodiversity Institute ^a^ | Blast in Genbank (Cover %, Identity %) ^b^ | Source | Isolation place |
| --- | --- | --- | --- | --- | --- | --- |
| 1 | A165-2 | MH062996 | *Cladophialophora boppii* (0.8 % difference) | *cladophialophora boppii（*C84%, I99%） | Haizhu,Guangzhou,China | Kitchen/water tank |
| 2 | A182-1 | MH062997 | *Cladophialophora boppii* (0.8 % difference) | *cladophialophora boppii*（C84%, I99%） | Yuexiu,Guangzhou,China | Kitchen/water tank |
| 3 | A184-2 | MH062998 | *Cladophialophora immunda* | *Cladophialophora immunda*（C77%, I100%） | Yuexiu,Guangzhou,China | Kitchen/Chopping board |
| 4 | A150-1 | MH062995 | *Cladophialophora boppii* (2 % difference) | *cladophialophora boppii*（C78%, I99%） | Haizhu,Guangzhou,China | Bathroom/wall |
| 5 | A415-1 | MH063017 | *Cladosporium halotolerans* | *Cladosporium halotolerans*（C99%, I99%） | Baiyun,Guangzhou,China | Bathroom/soap box |
| 6 | A216-1 | MH063008 | *Cladosporium halotolerans* | *Cladosporium halotolerans*（C100%, I99%） | Haizhu,Guangzhou,China | Bathroom/wall |
| 7 | A226-1 | MH063009 | *Cladosporium halotolerans* | *Cladosporium halotolerans*（C100%, I98%） | Haizhu,Guangzhou,China | Refrigerator/rubber seal |
| 8 | A145-1 | MH063006 | *Cladosporium halotolerans* | *Cladosporium halotolerans*（C100%, I99%） | Haizhu,Guangzhou,China | Refrigerator/rubber seal |
| 9 | A1-1 | MH062999 | *Cladosporium halotolerans* | *Cladosporium halotolerans*（C100%, I99%） | Haizhu,Guangzhou,China | Refrigerator/rubber seal |
| 10 | A11-3 | MH063000 | *Cladosporium halotolerans* | *Cladosporium halotolerans*（C99%, I100%） | Haizhu,Guangzhou,China | Wash machine/rubber seal |
| 11 | A58-1 | MH063001 | *Cladosporium halotolerans* | *Cladosporium halotolerans*（C100%, I100%） | Haizhu,Guangzhou,China | Wash machine/rubber seal |
| 12 | A90-1 | MH063005 | *Cladosporium halotolerans* | *Cladosporium halotolerans*（C100%, I99%） | Haizhu,Guangzhou,China | Refrigerator/rubber seal |
| 13 | A65-1 | MH063002 | *Cladosporium halotolerans* | *Cladosporium halotolerans*（C100%, I99%） | Haizhu,Guangzhou,China | Bathroom/mirror |
| 14 | A67-1 | MH063003 | *Cladosporium halotolerans* | *Cladosporium halotolerans*（C100%, I99%） | Haizhu,Guangzhou,China | Refrigerator/rubber seal |
| 15 | A71-2 | MH063004 | *Cladosporium halotolerans* | *Cladosporium halotolerans*（C99%, I99%） | Haizhu,Guangzhou,China | Kitchen/water tank |
| 16 | Y8-1 | MH063019 | *Cladosporium halotolerans* | *Cladosporium halotolerans*（C100%, I99%） | Haizhu,Guangzhou,China | Bathroom/wall |
| 17 | A287-1 | MH063014 | *Cladosporium halotolerans* | *Cladosporium halotolerans*（C99%, I99%） | Haizhu,Guangzhou,China | Kitchen/Chopping board |
| 18 | A250-2 | MH063010 | *Cladosporium halotolerans* | *Cladosporium halotolerans*（C100%, I99%） | Liwan,Guangzhou,China | Bathroom/wall |
| 19 | A260-1 | MH063011 | *Cladosporium halotolerans* | *Cladosporium halotolerans*（C100%, I99%） | Liwan,Guangzhou,China | Bathroom/washbasin |
| 20 | A268-1 | MH063012 | *Cladosporium halotolerans* | *Cladosporium halotolerans*（C100%, I99%） | Liwan,Guangzhou,China | Refrigerator/rubber seal |
| 21 | A369-1 | MH063015 | *Cladosporium halotolerans* | *Cladosporium halotolerans*（C99%, I99%） | Liwan,Guangzhou,China | Refrigerator/rubber seal |
| 22 | A389-1 | MH063016 | *Cladosporium halotolerans* | *Cladosporium halotolerans*（C100%, I99%） | Liwan,Guangzhou,China | Refrigerator/rubber seal |
| 23 | A441-1 | MH063018 | *Cladosporium halotolerans* | *Cladosporium halotolerans*（C100%, I99%） | Yuexiu,Guangzhou,China | Kitchen/water ladle |
| 24 | A185-3 | MH063013 | *Cladosporium halotolerans* | *Cladosporium halotolerans*（C99%, I99%） | Yuexiu,Guangzhou,China | Refrigerator/rubber seal |
| 25 | A193-1 | MH063007 | *Cladosporium halotolerans* | *Cladosporium halotolerans*（C100%, I99%） | Yuexiu,Guangzhou,China | Water dispenser |
| 26 | A228-1 | MH063027 | *Cladosporium irritans* (0.7% difference) | *Toxicocladosporium banksiae* (C100%, I96%) | Haizhu,Guangzhou,China | Refrigerator/rubber seal |
| 27 | A40-2 | MH063022 | *Cladosporium irritans* (0.7% difference) | *Toxicocladosporium banksiae* (C100%, I96%) | Haizhu,Guangzhou,China | Refrigerator/rubber seal |
| 28 | A488-2 | MH063032 | *Cladosporium irritans* (0.7% difference) | *Toxicocladosporium banksiae* (C100%, I96%) | Haizhu,Guangzhou,China | Kitchen/wall |
| 29 | A6-1 | MH063021 | *Cladosporium irritans* (0.7% difference) | *Toxicocladosporium banksiae* (C100%, I96%) | Haizhu,Guangzhou,China | Kitchen/cooking bench |
| 30 | A229-1 | MH063028 | *Cladosporium irritans* (0.7% difference) | *Toxicocladosporium banksiae* (C100%, I96%) | Liwan,Guangzhou,China | Refrigerator/rubber seal |
| 31 | A248-1 | MH063029 | *Cladosporium irritans* (0.7% difference) | *Toxicocladosporium banksiae* (C100%, I96%) | Liwan,Guangzhou,China | Kitchen/wall |
| 32 | A253-1 | MH063030 | *Cladosporium irritans* (0.7% difference) | *Toxicocladosporium banksiae* (C100%, I96%) | Liwan,Guangzhou,China | Bathroom/soap box |
| 33 | A401-2 | MH063031 | *Cladosporium irritans* (0.7% difference) | *Toxicocladosporium banksiae* (C100%, I96%) | Liwan,Guangzhou,China | Kitchen/Sterilizing cabinet |
| 34 | A105-1 | MH063020 | *Cladosporium irritans* (0.7% difference) | *Toxicocladosporium banksiae* (C100%, I96%) | Tianhe,Guangzhou,China | Kitchen/water tank |
| 35 | A112-2 | MH063023 | *Cladosporium irritans* (0.7% difference) | *Toxicocladosporium banksiae* (C100%, I96%) | Tianhe,Guangzhou,China | Refrigerator/rubber seal |
| 36 | A129-2 | MH063024 | *Cladosporium irritans* (0.7% difference) | *Toxicocladosporium banksiae* (C100%, I96%) | Tianhe,Guangzhou,China | Wash machine/rubber seal |
| 37 | A135-1 | MH063025 | *Cladosporium irritans* (0.7% difference) | *Toxicocladosporium banksiae* (C100%, I96%) | Tianhe,Guangzhou,China | Refrigerator/rubber seal |
| 38 | A197-1 | MH063026 | *Cladosporium irritans* (0.7% difference) | *Toxicocladosporium banksiae* (C100%, I96%) | Yuexiu,Guangzhou,China | Refrigerator/rubber seal |
| 39 | A41-3 | MH063033 | *Cladosporium oxysporum/tenuissimum* | *Cladosporium sp.* (C100%, I100%) | Haizhu,Guangzhou,China | Refrigerator/rubber seal |
| 40 | A42-3 | MH063034 | *Cladosporium oxysporum/tenuissimum* | *Cladosporium sp.* (C100%, I100%) | Haizhu,Guangzhou,China | Bathroom/washbasin |
| 41 | Y2-2 | MH063035 | *Cladosporium oxysporum/tenuissimum* | *Cladosporium sp.* (C99%, I99%) | Haizhu,Guangzhou,China | Wash machine/rubber seal |
| 42 | A324-1 | MH063040 | *Cyphellophora fusarioides* (1% difference) | *Cyphellophora laciniata* (C95%, I97%) | Haizhu,Guangzhou,China | Bathroom/washbasin |
| 43 | A157-1 | MH063037 | *Cyphellophora fusarioides* (1% difference) | *Cyphellophora laciniata* (C95%, I97%) | Haizhu,Guangzhou,China | Bathroom/washbasin |
| 44 | A244-1 | MH063038 | *Cyphellophora fusarioides* (1% difference) | *Cyphellophora laciniata* (C95%, I97%) | Liwan,Guangzhou,China | Bathroom/soap box |
| 45 | A245-1 | MH063039 | *Cyphellophora fusarioides* (1% difference) | *Cyphellophora laciniata* (C95%, I97%) | Liwan,Guangzhou,China | Bathroom/washbasin |
| 46 | A108-2 | MH063036 | *Cyphellophora fusarioides* (1% difference) | *Cyphellophora laciniata* (C95%, I97%) | Tianhe,Guangzhou,China | Wash machine/rubber seal |
| 47 | A174-3 | MH063041 | *Cyphellophora pluriseptata* (4% difference) | *Cyphellophora laciniata* (C89%, I91%) | Haizhu,Guangzhou,China | Bathroom/wall |
| 48 | A184-4 | MH063042 | *Cyphellophora pluriseptata* (4% difference) | *Cyphellophora laciniata* (C89%, I91%) | Yuexiu,Guangzhou,China | Kitchen/Chopping board |
| 49 | A408-1 | MH063052 | *Exophiala alcalophila* | *Exophiala alcalophila* (C80%, I99%) | Baiyun,Guangzhou,China | Bathroom/wall |
| 50 | A410-1 | MH063053 | *Exophiala alcalophila* | *Exophiala alcalophila* (C80%, I99%) | Baiyun,Guangzhou,China | Bathroom/washbasin |
| 51 | A427-1 | MH063055 | *Exophiala alcalophila* | *Exophiala alcalophila* (C80%, I99%) | Baiyun,Guangzhou,China | Kitchen/water tank |
| 52 | A414-1 | MH063054 | *Exophiala alcalophila* | *Exophiala alcalophila* (C80%, I99%) | Baiyun,Guangzhou,China | Bathroom/washbasin |
| 53 | A148-1 | MH063050 | *Exophiala alcalophila* | *Exophiala alcalophila* (C80%, I99%) | Haizhu,Guangzhou,China | Bathroom/washbasin |
| 54 | A48-1 | MH063045 | *Exophiala alcalophila* | *Exophiala alcalophila* (C80%, I99%) | Haizhu,Guangzhou,China | Bathroom/wall |
| 55 | A51-1 | MH063046 | *Exophiala alcalophila* | *Exophiala alcalophila* (C80%, I99%) | Haizhu,Guangzhou,China | bathroom/washbasin |
| 56 | A18-1 | MH063043 | *Exophiala alcalophila* | *Exophiala alcalophila* (C95%, I99%) | Haizhu,Guangzhou,China | Bathroom/wall |
| 57 | A70-1 | MH063047 | *Exophiala alcalophila* | *Exophiala alcalophila* (C80%, I99%) | Haizhu,Guangzhou,China | Bathroom/washbasin |
| 58 | A303-1 | MH063051 | *Exophiala alcalophila* | *Exophiala alcalophila* (C80%, I99%) | Haizhu,Guangzhou,China | Kitchen/water tank |
| 59 | A34-1 | MH063044 | *Exophiala alcalophila* | *Exophiala alcalophila* (C80%, I99%) | Haizhu,Guangzhou,China | Bathroom/soap box |
| 60 | A138-1 | MH063048 | *Exophiala alcalophila* | *Exophiala alcalophila* (C80%, I99%) | Yuexiu,Guangzhou,China | Kitchen/water tank |
| 61 | A141-1 | MH063049 | *Exophiala alcalophila* | *Exophiala alcalophila* (C80%, I99%) | Yuexiu,Guangzhou,China | Bathroom/toothbrush cup |
| 62 | A435-1 | MH063063 | *Exophiala cancerae* | *Exophiala salmonis* (C79%, I99%) | Baiyun,Guangzhou,China | Bathroom/washbasin |
| 63 | A350-3 | MH063062 | *Exophiala cancerae* | *Exophiala salmonis* (C79%, I99%) | Haizhu,Guangzhou,China | Bathroom/washbasin |
| 64 | A147-1 | MH063058 | *Exophiala cancerae* | *Exophiala salmonis* (C79%, I99%) | Haizhu,Guangzhou,China | Bathroom/washbasin |
| 65 | A181-1 | MH063059 | *Exophiala cancerae* | *Exophiala salmonis* (C79%, I99%) | Haizhu,Guangzhou,China | Bathroom/toothbrush cup |
| 66 | A5-1 | MH063057 | *Exophiala cancerae* | *Exophiala salmonis* (C79%, I99%) | Haizhu,Guangzhou,China | Kitchen/water tank |
| 67 | Y15-1 | MH063065 | *Exophiala cancerae* | *Exophiala salmonis* (C79%, I99%) | Haizhu,Guangzhou,China | Bathroom/toothbrush cup |
| 68 | Y6-1 | MH063064 | *Exophiala cancerae* | *Exophiala salmonis* (C79%, I99%) | Haizhu,Guangzhou,China | Bathroom/wall |
| 69 | A238-2 | MH063061 | *Exophiala cancerae* | *Exophiala salmonis* (C79%, I99%) | Liwan,Guangzhou,China | Bathroom/toothbrush cup |
| 70 | A186-2 | MH063060 | *Exophiala cancerae* | *Exophiala salmonis* (C79%, I99%) | Yuexiu,Guangzhou,China | Refrigerator/rubber seal |
| 71 | A457-4 | MH063069 | *Exophiala dermatitidis* | *Exophiala dermatitidis* (C84%, I99%) | Haizhu,Guangzhou,China | Kitchen/Chopping board |
| 72 | A486-2 | MH063070 | *Exophiala dermatitidis* | *Exophiala dermatitidis* (C84%, I99%) | Haizhu,Guangzhou,China | Kitchen/Chopping board |
| 73 | A100-1 | MH063066 | *Exophiala dermatitidis* | *Exophiala dermatitidis* (C83%, I100%) | Haizhu,Guangzhou,China | Refrigerator/rubber seal |
| 74 | A235-2 | MH063068 | *Exophiala dermatitidis* | *Exophiala dermatitidis* (C82%, I100%) | Liwan,Guangzhou,China | Kitchen/Chopping board |
| 75 | A184-5 | MH063067 | *Exophiala dermatitidis* | *Exophiala dermatitidis* (C83%, I199%) | Yuexiu,Guangzhou,China | Kitchen/Chopping board |
| 76 | A350-1 | MH063080 | *Exophiala equina* | *Exophiala sp.* (C79%, I99%) | Haizhu,Guangzhou,China | Bathroom/washbasin |
| 77 | A142-1 | MH063074 | *Exophiala equina* | *Exophiala sp.* (C79%, I99%) | Haizhu,Guangzhou,China | Kitchen/water tank |
| 78 | A174-1 | MH063075 | *Exophiala equina* | *Exophiala sp.* (C79%, I99%) | Haizhu,Guangzhou,China | Bathroom/wall |
| 79 | A18-2 | MH063071 | *Exophiala equina* | *Exophiala sp.* (C79%, I99%) | Haizhu,Guangzhou,China | Bathroom/wall |
| 80 | A31-1 | MH063072 | *Exophiala equina* | *Exophiala sp.* (C79%, I99%) | Haizhu,Guangzhou,China | Bathroom/washbasin |
| 81 | A313-2 | MH063079 | *Exophiala equina* | *Exophiala sp.* (C79%, I99%) | Haizhu,Guangzhou,China | Bathroom/washbasin |
| 82 | A249-1 | MH063078 | *Exophiala equina* | *Exophiala sp.* (C79%, I99%) | Liwan,Guangzhou,China | Kitchen/water tank |
| 83 | A190-1 | MH063077 | *Exophiala equina* | *Exophiala sp.* (C79%, I99%) | Yuexiu,Guangzhou,China | Bathroom/wall |
| 84 | A36-2-1 | MH063073 | *Exophiala equina* | *Exophiala sp.* (C79%, I99%) | Haizhu,Guangzhou,China | Kitchen/Chopping board |
| 85 | A423-1 | MH063098 | *Exophiala lecanii-corni* | *Exophiala lecanii-corni* (C81%, I99%) | Baiyun,Guangzhou,China | Kitchen/water tank |
| 86 | A159-1 | MH063088 | *Exophiala lecanii-corni* | *Exophiala lecanii-corni* (C81%, I99%) | Haizhu,Guangzhou,China | Bathroom/wall |
| 87 | A38-1 | MH063083 | *Exophiala lecanii-corni* | *Exophiala lecanii-corni* (C82%, I99%) | Haizhu,Guangzhou,China | Kitchen/water tank |
| 88 | A12-1 | MH063082 | *Exophiala lecanii-corni* | *Exophiala lecanii-corni* (C82%, I99%) | Haizhu,Guangzhou,China | Bathroom/wall |
| 89 | A308-1 | MH063093 | *Exophiala lecanii-corni* | *Exophiala lecanii-corni* (C80%, I99%) | Haizhu,Guangzhou,China | Bathroom/washbasin |
| 90 | A310-1 | MH063094 | *Exophiala lecanii-corni* | *Exophiala lecanii-corni* (C81%, I99%) | Haizhu,Guangzhou,China | Bathroom/wall |
| 91 | Y14-1 | MH063099 | *Exophiala lecanii-corni* | *Exophiala lecanii-corni* (C99%, I99%) | Haizhu,Guangzhou,China | Bathroom/toothbrush cup |
| 92 | A314-3 | MH063095 | *Exophiala lecanii-corni* | *Exophiala lecanii-corni* (C81%, I99%) | Haizhu,Guangzhou,China | Bathroom/toothbrush cup |
| 93 | A315-1 | MH063096 | *Exophiala lecanii-corni* | *Exophiala lecanii-corni* (C81%, I99%) | Haizhu,Guangzhou,China | Bathroom/wall |
| 94 | A292-2 | MH063092 | *Exophiala lecanii-corni* | *Exophiala lecanii-corni* (C81%, I99%) | Haizhu,Guangzhou,China | Bathroom/toothbrush cup |
| 95 | A238-3 | MH063089 | *Exophiala lecanii-corni* | *Exophiala lecanii-corni* (C82%, I99%) | Liwan,Guangzhou,China | Bathroom/toothbrush cup |
| 96 | A265-1 | MH063090 | *Exophiala lecanii-corni* | *Exophiala lecanii-corni* (C81%, I99%) | Liwan,Guangzhou,China | Bathroom/toothbrush cup |
| 97 | A266-1 | MH063091 | *Exophiala lecanii-corni* | *Exophiala lecanii-corni* (C81%, I99%) | Liwan,Guangzhou,China | Kitchen/water ladle |
| 98 | A379-1 | MH063097 | *Exophiala lecanii-corni* | *Exophiala lecanii-corni* (C81%, I99%) | Liwan,Guangzhou,China | Kitchen/water tank |
| 99 | A109-1 | MH063084 | *Exophiala lecanii-corni* | *Exophiala lecanii-corni* (C82%, I99%) | Tianhe,Guangzhou,China | Bathroom/washbasin |
| 100 | A111-1 | MH063085 | *Exophiala lecanii-corni* | *Exophiala lecanii-corni* (C82%, I99%) | Tianhe,Guangzhou,China | Bathroom/washbasin |
| 101 | A121-2 | MH063086 | *Exophiala lecanii-corni* | *Exophiala lecanii-corni* (C82%, I99%) | Tianhe,Guangzhou,China | Bathroom/washbasin |
| 102 | A127-1 | MH063087 | *Exophiala lecanii-corni* | *Exophiala lecanii-corni* (C81%, I99%) | Tianhe,Guangzhou,China | Kitchen/Chopping board |
| 103 | A218-1 | MH063100 | *Exophiala mesophila* | *Exophiala mesophila* (C82%, I99%) | Haizhu,Guangzhou,China | Bathroom/washbasin |
| 104 | A437-1 | MH063101 | *Exophiala mesophila* | *Exophiala mesophila* (C82%, I99%) | Yuexiu,Guangzhou,China | Bathroom/washbasin |
| 105 | A444-1 | MH063102 | *Exophiala mesophila* | *Exophiala mesophila* (C83%, I99%) | Yuexiu,Guangzhou,China | Kitchen/water tank |
| 106 | A457-2 | MH063113 | *Exophiala oligosperma* | *Exophiala oligosperma* (C85%, I99%) | Haizhu,Guangzhou,China | Kitchen/Chopping board |
| 107 | A475-1 | MH063114 | *Exophiala oligosperma* | *Exophiala oligosperma* (C87%, I99%) | Haizhu,Guangzhou,China | Kitchen/water tank |
| 108 | A476-1 | MH063115 | *Exophiala oligosperma* | *Exophiala oligosperma* (C87%, I99%) | Haizhu,Guangzhou,China | Kitchen/Chopping board |
| 109 | A81-3 | MH063112 | *Exophiala oligosperma* | *Exophiala oligosperma* (C87%, I99%) | Haizhu,Guangzhou,China | Kitchen/Chopping board |
| 110 | A302-1 | MH063111 | *Exophiala oligosperma* | *Exophiala oligosperma* (C87%, I99%) | Haizhu,Guangzhou,China | Kitchen/water tank |
| 111 | A36-1 | MH063106 | *Exophiala oligosperma* | *Exophiala oligosperma* (C87%, I99%) | Haizhu,Guangzhou,China | Kitchen/Chopping board |
| 112 | A71-4 | MH063107 | *Exophiala oligosperma* | *Exophiala oligosperma* (C87%, I99%) | Haizhu,Guangzhou,China | Kitchen/water tank |
| 113 | A231-2 | MH063109 | *Exophiala oligosperma* | *Exophiala oligosperma* (C87%, I99%) | Liwan,Guangzhou,China | Refrigerator/rubber seal |
| 114 | A235-1 | MH063110 | *Exophiala oligosperma* | *Exophiala oligosperma* (C87%, I99%) | Liwan,Guangzhou,China | Kitchen/Chopping board |
| 115 | A199-3 | MH063108 | *Exophiala oligosperma* | *Exophiala oligosperma* (C87%, I99%) | Yuexiu,Guangzhou,China | Refrigerator/rubber seal |
| 116 | Y2-1 | MH063117 | *Exophiala sp.* | *Exophiala alcalophila* (C83%, I99%) | Haizhu,Guangzhou,China | Wash machine/rubber seal |
| 117 | A362-1 | MH063116 | *Exophiala sp.* | *Exophiala alcalophila* (C83%, I99%) | Liwan,Guangzhou,China | Refrigerator/rubber seal |
| 118 | A465-2 | MH063056 | *Exophiala aquamarina* (10% difference) | *Exophiala sp.* (C76%, I87%) | Haizhu,Guangzhou,China | Bathroom/washbasin |
| 119 | A183-1 | MH063076 | *Exophiala equina* (11% difference) | *Chaetothyriales sp.* (C95%, I92%) | Yuexiu,Guangzhou,China | Kitchen/water tank |
| 120 | A287-4 | MH063103 | *Exophiala nishimurae* (5% difference) | *Exophiala sp.* (C100%, I91%) | Haizhu,Guangzhou,China | Kitchen/Chopping board |
| 121 | A357-1 | MH063104 | *Exophiala nishimurae* (5% difference) | *Exophiala sp.* (C100%, I91%) | Liwan,Guangzhou,China | Kitchen/water tank |
| 122 | A486-1 | MH063105 | *Exophiala nishimurae* (5% difference) | *Exophiala sp.* (C100%, I91%) | Haizhu,Guangzhou,China | Kitchen/Chopping board |
| 123 | A365-1 | MH063081 | *Exophiala jeanselmei* (5% difference) | *Exophiala sp.* (C100%, I93%) | Liwan,Guangzhou,China | Kitchen/Chopping board |
| 124 | A25-1 | MH063118 | *Exophiala xenobiotica* | *Exophiala xenobiotica* (C83%, I100%) | Haizhu,Guangzhou,China | Kitchen/water tank |
| 125 | A184-3 | MH063119 | *Exophiala xenobiotica* | *Exophiala xenobiotica* (C83%, I100%) | Yuexiu,Guangzhou,China | Kitchen/Chopping board |
| 126 | A340-2 | MH063121 | *Hortaea werneckii*（8% difference ) | *Acremonium sp.* (C80%, I99%) | Haizhu,Guangzhou,China | Wash machine/rubber seal |
| 127 | A96-1 | MH063120 | *Hortaea werneckii*（8% difference ) | *Acremonium sp.* (C81%, I99%) | Haizhu,Guangzhou,China | Kitchen/Chopping board |
| 128 | A6-2 | MH063122 | *Hortaea werneckii* | *Hortaea werneckii* (C93%, I99%) | Haizhu,Guangzhou,China | Kitchen/cooking bench |
| 129 | A370-1 | MH063125 | *Hortaea werneckii* | *Hortaea werneckii* (C93%, I98%) | Liwan,Guangzhou,China | Refrigerator/rubber seal |
| 130 | A372-3 | MH063126 | *Hortaea werneckii* | *Hortaea werneckii* (C93%, I98%) | Liwan,Guangzhou,China | Bathroom/washbasin |
| 131 | A401-1 | MH063124 | *Hortaea werneckii* | *Hortaea werneckii* (C93%, I99%) | Liwan,Guangzhou,China | Kitchen/Sterilizing cabinet |
| 132 | A197-2 | MH063123 | *Hortaea werneckii* | *Hortaea werneckii* (C91%, I99%) | Yuexiu,Guangzhou,China | Refrigerator/rubber seal |
| 133 | A217-2 | MH063149 | *Knufia epidermidis* | *Knufia epidermidis*（C79%, I99%) | Haizhu,Guangzhou,China | Bathroom/sprinklers |
| 134 | A220-2 | MH063150 | *Knufia epidermidis* | *Knufia epidermidis*（C79%, I99%) | Haizhu,Guangzhou,China | Bathroom/wall |
| 135 | A337-1 | MH063157 | *Knufia epidermidis* | *Knufia epidermidis*（C79%, I99%) | Haizhu,Guangzhou,China | Bathroom/wall |
| 136 | A353-2 | MH063158 | *Knufia epidermidis* | *Knufia epidermidis*（C79%, I99%) | Haizhu,Guangzhou,China | Bathroom/wall |
| 137 | A142-2 | MH063142 | *Knufia epidermidis* | *Knufia epidermidis*（C79%, I99%) | Haizhu,Guangzhou,China | Kitchen/water tank |
| 138 | A162-1 | MH063143 | *Knufia epidermidis* | *Knufia epidermidis*（C79%, I99%) | Haizhu,Guangzhou,China | Bathroom/wall |
| 139 | A165-4 | MH063144 | *Knufia epidermidis* | *Knufia epidermidis*（C79%, I99%) | Haizhu,Guangzhou,China | Kitchen/water tank |
| 140 | A172-2 | MH063145 | *Knufia epidermidis* | *Knufia epidermidis*（C79%, I99%) | Haizhu,Guangzhou,China | Bathroom/washbasin |
| 141 | A175-2 | MH063146 | *Knufia epidermidis* | *Knufia epidermidis*（C79%, I99%) | Haizhu,Guangzhou,China | Bathroom/wall |
| 142 | A180-1 | MH063147 | *Knufia epidermidis* | *Knufia epidermidis*（C79%, I99%) | Haizhu,Guangzhou,China | Bathroom/wall |
| 143 | A181-5 | MH063148 | *Knufia epidermidis* | *Knufia epidermidis*（C79%, I99%) | Haizhu,Guangzhou,China | Bathroom/toothbrush cup |
| 144 | A43-1 | MH063132 | *Knufia epidermidis* | *Knufia epidermidis*（C79%, I99%) | Haizhu,Guangzhou,China | Bathroom/brush |
| 145 | A10-2 | MH063128 | *Knufia epidermidis* | *Knufia epidermidis*（C80%, I99%) | Haizhu,Guangzhou,China | Wash machine/rubber seal |
| 146 | A12-3 | MH063129 | *Knufia epidermidis* | *Knufia epidermidis*（C79%, I99%) | Haizhu,Guangzhou,China | Bathroom/wall |
| 147 | A3-1 | MH063127 | *Knufia epidermidis* | *Knufia epidermidis*（C79%, I99%) | Haizhu,Guangzhou,China | Bathroom/toothbrush cup |
| 148 | A30-3 | MH063130 | *Knufia epidermidis* | *Knufia epidermidis*（C79%, I99%) | Haizhu,Guangzhou,China | Bathroom/washbasin |
| 149 | A33-3 | MH063131 | *Knufia epidermidis* | *Knufia epidermidis*（C79%, I99%) | Haizhu,Guangzhou,China | Wash machine/rubber seal |
| 150 | A88-4 | MH063133 | *Knufia epidermidis* | *Knufia epidermidis*（C80%, I99%) | Haizhu,Guangzhou,China | Kitchen/water tank |
| 151 | A93-1 | MH063134 | *Knufia epidermidis* | *Knufia epidermidis*（C83%, I99%) | Haizhu,Guangzhou,China | Bathroom/wall |
| 152 | A97-1 | MH063135 | *Knufia epidermidis* | *Knufia epidermidis*（C79%, I99%) | Haizhu,Guangzhou,China | Bathroom/washbasin |
| 153 | A99-1 | MH063136 | *Knufia epidermidis* | *Knufia epidermidis*（C79%, I99%) | Haizhu,Guangzhou,China | Bathroom/sprinklers |
| 154 | A294-1 | MH063155 | *Knufia epidermidis* | *Knufia epidermidis*（C79%, I99%) | Haizhu,Guangzhou,China | Bathroom/washbasin |
| 155 | A297-1 | MH063156 | *Knufia epidermidis* | *Knufia epidermidis*（C79%, I99%) | Haizhu,Guangzhou,China | Kitchen/water tank |
| 156 | Y12-1 | MH063163 | *Knufia epidermidis* | *Knufia epidermidis*（C98%, I100%) | Haizhu,Guangzhou,China | Bathroom/washbasin |
| 157 | Y4-1 | MH063162 | *Knufia epidermidis* | *Knufia epidermidis*（C99%, I99%) | Haizhu,Guangzhou,China | Wash machine/rubber seal |
| 158 | A232-1 | MH063151 | *Knufia epidermidis* | *Knufia epidermidis*（C79%, I99%) | Liwan,Guangzhou,China | Kitchen/wall |
| 159 | A236-1 | MH063152 | *Knufia epidermidis* | *Knufia epidermidis*（C80%, I99%) | Liwan,Guangzhou,China | Bathroom/wall |
| 160 | A252-2 | MH063153 | *Knufia epidermidis* | *Knufia epidermidis*（C79%, I99%) | Liwan,Guangzhou,China | Bathroom/wall |
| 161 | A261-1 | MH063154 | *Knufia epidermidis* | *Knufia epidermidis*（C79%, I99%) | Liwan,Guangzhou,China | Bathroom/wall |
| 162 | A386-2 | MH063159 | *Knufia epidermidis* | *Knufia epidermidis*（C79%, I99%) | Liwan,Guangzhou,China | Bathroom/wall |
| 163 | A394-2 | MH063160 | *Knufia epidermidis* | *Knufia epidermidis*（C79%, I99%) | Liwan,Guangzhou,China | Bathroom/wall |
| 164 | A120-1 | MH063137 | *Knufia epidermidis* | *Knufia epidermidis*（C79%, I99%) | Tianhe,Guangzhou,China | Bathroom/door |
| 165 | A128-1 | MH063138 | *Knufia epidermidis* | *Knufia epidermidis*（C79%, I99%) | Tianhe,Guangzhou,China | Bathroom/wall |
| 166 | A130-1 | MH063139 | *Knufia epidermidis* | *Knufia epidermidis*（C79%, I99%) | Tianhe,Guangzhou,China | Bathroom/floor |
| 167 | A134-1 | MH063140 | *Knufia epidermidis* | *Knufia epidermidis*（C79%, I99%) | Tianhe,Guangzhou,China | Kitchen/water tank |
| 168 | A138-2 | MH063141 | *Knufia epidermidis* | *Knufia epidermidis*（C79%, I99%) | Yuexiu,Guangzhou,China | Kitchen/water tank |
| 169 | A443-1 | MH063161 | *Knufia epidermidis* | *Knufia epidermidis*（C79%, I99%) | Yuexiu,Guangzhou,China | Kitchen/water tank |
| 170 | A403-1 | MH063166 | *Ochroconis humicola* (0.2% difference) | *Ochroconis mirabilis*（C78%, I99%) | Baiyun,Guangzhou,China | Bathroom/wall |
| 171 | A413-2 | MH063168 | *Ochroconis humicola* (0.2% difference) | *Ochroconis mirabilis*（C78%, I99%) | Baiyun,Guangzhou,China | Bathroom/washbasin |
| 172 | A48-3 | MH063164 | *Ochroconis humicola* (0.2% difference) | *Ochroconis mirabilis*（C78%, I99%) | Haizhu,Guangzhou,China | Bathroom/wall |
| 173 | A19-3-2 | MH063167 | *Ochroconis humicola* (0.2% difference) | *Ochroconis mirabilis*（C77%, I99%) | Haizhu,Guangzhou,China | Bathroom/wall |
| 174 | Y6-2 | MH063169 | *Ochroconis humicola* (0.2% difference) | *Ochroconis mirabilis*（C100%, I99%) | Haizhu,Guangzhou,China | Bathroom/wall |
| 175 | A237-2 | MH063165 | *Ochroconis humicola* (0.2% difference) | *Ochroconis mirabilis*（C78%, I99%) | Liwan,Guangzhou,China | Bathroom/wall |
| 176 | A408-3 | MH063198 | *Ochroconis musae* | *Ochroconis humicola*（C85%, I99%) | Baiyun,Guangzhou,China | Bathroom/wall |
| 177 | A428-6 | MH063199 | *Ochroconis musae* | *Ochroconis humicola*（C85%, I99%) | Baiyun,Guangzhou,China | Kitchen/water tank |
| 178 | A155-5 | MH063188 | *Ochroconis musae* | *Ochroconis humicola*（C85%, I99%) | Haizhu,Guangzhou,China | Kitchen/water tank |
| 179 | A175-4 | MH063189 | *Ochroconis musae* | *Ochroconis humicola*（C85%, I99%) | Haizhu,Guangzhou,China | Bathroom/wall |
| 180 | A177-2-1 | MH063191 | *Ochroconis musae* | *Ochroconis humicola*（C85%, I99%) | Haizhu,Guangzhou,China | Kitchen/water tank |
| 181 | A40-1 | MH063175 | *Ochroconis musae* | *Ochroconis humicola*（C85%, I99%) | Haizhu,Guangzhou,China | Refrigerator/rubber seal |
| 182 | A42-2 | MH063176 | *Ochroconis musae* | *Ochroconis humicola*（C85%, I99%) | Haizhu,Guangzhou,China | Bathroom/washbasin |
| 183 | A49-1 | MH063177 | *Ochroconis musae* | *Ochroconis humicola*（C85%, I99%) | Haizhu,Guangzhou,China | Bathroom/wall |
| 184 | A11-2 | MH063172 | *Ochroconis musae* | *Ochroconis humicola*（C85%, I99%) | Haizhu,Guangzhou,China | Wash machine/rubber seal |
| 185 | A12-2 | MH063190 | *Ochroconis musae* | *Ochroconis humicola*（C85%, I99%) | Haizhu,Guangzhou,China | Bathroom/wall |
| 186 | A5-2 | MH063170 | *Ochroconis musae* | *Ochroconis humicola*（C85%, I99%) | Haizhu,Guangzhou,China | Kitchen/water tank |
| 187 | A7-1 | MH063171 | *Ochroconis musae* | *Ochroconis humicola*（C85%, I99%) | Haizhu,Guangzhou,China | Bathroom/washbasin |
| 188 | A20-1 | MH063173 | *Ochroconis musae* | *Ochroconis humicola*（C85%, I99%) | Haizhu,Guangzhou,China | Kitchen/water tank |
| 189 | A32-2 | MH063174 | *Ochroconis musae* | *Ochroconis humicola*（C85%, I99%) | Haizhu,Guangzhou,China | Wash machine/rubber seal |
| 190 | A60-2 | MH063178 | *Ochroconis musae* | *Ochroconis humicola*（C85%, I99%) | Haizhu,Guangzhou,China | Bathroom/washbasin |
| 191 | A68-1 | MH063181 | *Ochroconis musae* | *Ochroconis humicola*（C85%, I99%) | Haizhu,Guangzhou,China | Wash machine/rubber seal |
| 192 | A70-2 | MH063182 | *Ochroconis musae* | *Ochroconis humicola*（C85%, I99%) | Haizhu,Guangzhou,China | Bathroom/washbasin |
| 193 | A97-3 | MH063184 | *Ochroconis musae* | *Ochroconis humicola*（C85%, I99%) | Haizhu,Guangzhou,China | Bathroom/washbasin |
| 194 | A63-2 | MH063179 | *Ochroconis musae* | *Ochroconis humicola*（C85%, I99%) | Haizhu,Guangzhou,China | Wash machine/rubber seal |
| 195 | A64-2 | MH063180 | *Ochroconis musae* | *Ochroconis humicola*（C85%, I99%) | Haizhu,Guangzhou,China | Bathroom/door |
| 196 | A296-3 | MH063196 | *Ochroconis musae* | *Ochroconis humicola*（C85%, I99%) | Haizhu,Guangzhou,China | Kitchen/water tank |
| 197 | A72-2 | MH063183 | *Ochroconis musae* | *Ochroconis humicola*（C85%, I99%) | Haizhu,Guangzhou,China | Kitchen/water tank |
| 198 | Y11-1 | MH063201 | *Ochroconis musae* | *Ochroconis humicola*（C85%, I99%) | Haizhu,Guangzhou,China | Bathroom/washbasin |
| 199 | A278-6 | MH063194 | *Ochroconis musae* | *Ochroconis humicola*（C85%, I99%) | Haizhu,Guangzhou,China | Kitchen/water tank |
| 200 | A292-1 | MH063195 | *Ochroconis musae* | *Ochroconis humicola*（C85%, I99%) | Haizhu,Guangzhou,China | Bathroom/toothbrush cup |
| 201 | A272-1 | MH063193 | *Ochroconis musae* | *Ochroconis humicola*（C85%, I99%) | Liwan,Guangzhou,China | Kitchen/water tank |
| 202 | A397-1 | MH063197 | *Ochroconis musae* | *Ochroconis humicola*（C85%, I99%) | Liwan,Guangzhou,China | Wash machine/rubber seal |
| 203 | A126-1 | MH063185 | *Ochroconis musae* | *Ochroconis humicola*（C85%, I99%) | Tianhe,Guangzhou,China | Kitchen/water tank |
| 204 | A127-3 | MH063186 | *Ochroconis musae* | *Ochroconis humicola*（C85%, I99%) | Tianhe,Guangzhou,China | Kitchen/Chopping board |
| 205 | A134-3 | MH063187 | *Ochroconis musae* | *Ochroconis humicola*（C85%, I99%) | Tianhe,Guangzhou,China | Kitchen/water tank |
| 206 | A447-5 | MH063200 | *Ochroconis musae* | *Ochroconis humicola*（C85%, I99%) | Yuexiu,Guangzhou,China | Kitchen/Chopping board |
| 207 | A200-1 | MH063192 | *Ochroconis musae* | *Ochroconis humicola*（C85%, I99%) | Yuexiu,Guangzhou,China | Bathroom/wall |
| 208 | A328-2 | MH063212 | *Phialophora europaea* | *Phialophora europaea*（C80%, I99%) | Haizhu,Guangzhou,China | Kitchen/water tank |
| 209 | A353-1 | MH063213 | *Phialophora europaea* | *Phialophora europaea*（C80%, I99%) | Haizhu,Guangzhou,China | Bathroom/wall |
| 210 | A165-5 | MH063208 | *Phialophora europaea* | *Phialophora europaea*（C80%, I99%) | Haizhu,Guangzhou,China | Kitchen/water tank |
| 211 | A457-7 | MH063219 | *Phialophora europaea* | *Phialophora europaea*（C80%, I99%) | Haizhu,Guangzhou,China | Kitchen/Chopping board |
| 212 | A14-1 | MH063205 | *Phialophora europaea* | *Phialophora europaea*（C80%, I99%) | Haizhu,Guangzhou,China | Bathroom/washbasin |
| 213 | A20-2 | MH063202 | *Phialophora europaea* | *Phialophora europaea*（C80%, I99%) | Haizhu,Guangzhou,China | Kitchen/water tank |
| 214 | A91-1 | MH063203 | *Phialophora europaea* | *Phialophora europaea*（C80%, I99%) | Haizhu,Guangzhou,China | Refrigerator/rubber seal |
| 215 | A299-1 | MH063204 | *Phialophora europaea* | *Phialophora europaea*（C80%, I99%) | Haizhu,Guangzhou,China | Kitchen/Chopping board |
| 216 | A72-4 | MH063206 | *Phialophora europaea* | *Phialophora europaea*（C80%, I99%) | Haizhu,Guangzhou,China | Kitchen/water tank |
| 217 | A235-4 | MH063211 | *Phialophora europaea* | *Phialophora europaea*（C80%, I99%) | Liwan,Guangzhou,China | Kitchen/Chopping board |
| 218 | A358-2 | MH063214 | *Phialophora europaea* | *Phialophora europaea*（C80%, I99%) | Liwan,Guangzhou,China | Kitchen/Chopping board |
| 219 | A397-2 | MH063215 | *Phialophora europaea* | *Phialophora europaea*（C80%, I99%) | Liwan,Guangzhou,China | Wash machine/rubber seal |
| 220 | A118-2 | MH063207 | *Phialophora europaea* | *Phialophora europaea*（C80%, I99%) | Tianhe,Guangzhou,China | Kitchen/Chopping board |
| 221 | A437-2 | MH063216 | *Phialophora europaea* | *Phialophora europaea*（C80%, I99%) | Yuexiu,Guangzhou,China | Bathroom/washbasin |
| 222 | A445-1 | MH063217 | *Phialophora europaea* | *Phialophora europaea*（C80%, I99%) | Yuexiu,Guangzhou,China | Bathroom/soap box |
| 223 | A447-4 | MH063218 | *Phialophora europaea* | *Phialophora europaea*（C80%, I99%) | Yuexiu,Guangzhou,China | Kitchen/Chopping board |
| 224 | A189-1 | MH063209 | *Phialophora europaea* | *Phialophora europaea*（C80%, I99%) | Yuexiu,Guangzhou,China | Bathroom/wall |
| 225 | A198-2 | MH063210 | *Phialophora europaea* | *Phialophora europaea*（C80%, I99%) | Yuexiu,Guangzhou,China | Refrigerator/rubber seal |
| 226 | A424-1 | MH063243 | *Phialophora oxyspora* | *Phialophora oxyspora*（C81%, I99%) | Baiyun,Guangzhou,China | Kitchen/water tank |
| 227 | A336-2 | MH063241 | *Phialophora oxyspora* | *Phialophora oxyspora*（C81%, I99%) | Haizhu,Guangzhou,China | Refrigerator/rubber seal |
| 228 | A155-3 | MH063226 | *Phialophora oxyspora* | *Phialophora oxyspora*（C81%, I99%) | Haizhu,Guangzhou,China | Kitchen/water tank |
| 229 | A165-6 | MH063227 | *Phialophora oxyspora* | *Phialophora oxyspora*（C81%, I99%) | Haizhu,Guangzhou,China | Kitchen/water tank |
| 230 | A173-1 | MH063228 | *Phialophora oxyspora* | *Phialophora oxyspora*（C81%, I99%) | Haizhu,Guangzhou,China | Bathroom/washbasin |
| 231 | A178-1 | MH063229 | *Phialophora oxyspora* | *Phialophora oxyspora*（C81%, I99%) | Haizhu,Guangzhou,China | Kitchen/water tank |
| 232 | A8-2 | MH063220 | *Phialophora oxyspora* | *Phialophora oxyspora*（C81%, I99%) | Haizhu,Guangzhou,China | Bathroom/washbasin |
| 233 | A79-3 | MH063223 | *Phialophora oxyspora* | *Phialophora oxyspora*（C81%, I99%) | Haizhu,Guangzhou,China | Kitchen/water tank |
| 234 | A97-2 | MH063221 | *Phialophora oxyspora* | *Phialophora oxyspora*（C81%, I99%) | Haizhu,Guangzhou,China | Bathroom/washbasin |
| 235 | A66-1 | MH063222 | *Phialophora oxyspora* | *Phialophora oxyspora*（C81%, I99%) | Haizhu,Guangzhou,China | Bathroom/washbasin |
| 236 | A296-1 | MH063238 | *Phialophora oxyspora* | *Phialophora oxyspora*（C81%, I99%) | Haizhu,Guangzhou,China | Kitchen/water tank |
| 237 | Y14-2 | MH063244 | *Phialophora oxyspora* | *Phialophora oxyspora*（C81%, I99%) | Haizhu,Guangzhou,China | Bathroom/toothbrush cup |
| 238 | A313-1 | MH063239 | *Phialophora oxyspora* | *Phialophora oxyspora*（C81%, I99%) | Haizhu,Guangzhou,China | Bathroom/washbasin |
| 239 | A317-3 | MH063240 | *Phialophora oxyspora* | *Phialophora oxyspora*（C81%, I99%) | Haizhu,Guangzhou,China | Kitchen/water tank |
| 240 | A278-1 | MH063236 | *Phialophora oxyspora* | *Phialophora oxyspora*（C81%, I99%) | Haizhu,Guangzhou,China | Kitchen/water tank |
| 241 | A293-1 | MH063237 | *Phialophora oxyspora* | *Phialophora oxyspora*（C81%, I99%) | Haizhu,Guangzhou,China | Bathroom/toothbrush cup |
| 242 | A238-1 | MH063230 | *Phialophora oxyspora* | *Phialophora oxyspora*（C81%, I99%) | Liwan,Guangzhou,China | Bathroom/toothbrush cup |
| 243 | A240-1 | MH063231 | *Phialophora oxyspora* | *Phialophora oxyspora*（C81%, I99%) | Liwan,Guangzhou,China | Kitchen/water tank |
| 244 | A249-2 | MH063232 | *Phialophora oxyspora* | *Phialophora oxyspora*（C81%, I99%) | Liwan,Guangzhou,China | Kitchen/water tank |
| 245 | A265-2 | MH063233 | *Phialophora oxyspora* | *Phialophora oxyspora*（C81%, I99%) | Liwan,Guangzhou,China | Bathroom/toothbrush cup |
| 246 | A266-2 | MH063234 | *Phialophora oxyspora* | *Phialophora oxyspora*（C81%, I99%) | Liwan,Guangzhou,China | Kitchen/water ladle |
| 247 | A271-2 | MH063235 | *Phialophora oxyspora* | *Phialophora oxyspora*（C81%, I99%) | Liwan,Guangzhou,China | Kitchen/water tank |
| 248 | A372-1 | MH063242 | *Phialophora oxyspora* | *Phialophora oxyspora*（C81%, I99%) | Liwan,Guangzhou,China | Bathroom/washbasin |
| 249 | A120-4 | MH063224 | *Phialophora oxyspora* | *Phialophora oxyspora*（C81%, I99%) | Tianhe,Guangzhou,China | Bathroom/door |
| 250 | A127-2 | MH063225 | *Phialophora oxyspora* | *Phialophora oxyspora*（C81%, I99%) | Tianhe,Guangzhou,China | Kitchen/Chopping board |
| 251 | A123-1 | MH063245 | *Phialophora verrucosa* | *Phialophora americana* (C83%, I99%) | Tianhe,Guangzhou,China | Kitchen/Chopping board |
| 252 | A427-3 | MH063251 | *Rhinocladiella similis* | *Rhinocladiella similis* (C84%, I99%) | Baiyun,Guangzhou,China | Kitchen/water tank |
| 253 | A430-1 | MH063252 | *Rhinocladiella similis* | *Rhinocladiella similis* (C82%, I99%) | Baiyun,Guangzhou,China | Kitchen/Chopping board |
| 254 | A426-1 | MH063250 | *Rhinocladiella similis* | *Rhinocladiella similis* (C82%, I99%) | Baiyun,Guangzhou,China | Kitchen/Chopping board |
| 255 | A54-1 | MH063246 | *Rhinocladiella similis* | *Rhinocladiella similis* (C84%, I99%) | Haizhu,Guangzhou,China | bathroom/washbasin |
| 256 | A277-1 | MH063249 | *Rhinocladiella similis* | *Rhinocladiella similis* (C82%, I99%) | Haizhu,Guangzhou,China | Kitchen/water tank |
| 257 | A231-1 | MH063248 | *Rhinocladiella similis* | *Rhinocladiella similis* (C83%, I99%) | Liwan,Guangzhou,China | Refrigerator/rubber seal |
| 258 | A196-1 | MH063247 | *Rhinocladiella similis* | *Rhinocladiella similis* (C82%, I99%) | Yuexiu,Guangzhou,China | Kitchen/water tank |
| 259 | A385-1 | MH063253 | *Veronaea japonica* (6 % difference) | *Chaetothyriales sp.* (C100%,I99%) | Liwan,Guangzhou,China | Kitchen/Chopping board |
| 260 | A406-1 | - | Unknown species | *Chaetothyriales* sp. (C95%,I90%) | Bai Yun, Guangzhou, China | Kitchen/water tank |
| 261 | A404-2 | - | Unknown species | *Phialophora europaea*（C79%, I95%) | Bai Yun, Guangzhou, China | Bathroom/wall |
| 262 | A434-2 | - | Unknown species | *Phialophora europaea*（C80%, I95%) | Bai Yun, Guangzhou, China | Bathroom/toothbrush cup |
| 263 | A435-5 | - | Unknown species | *Phialophora europaea*（C80%, I95%) | Bai Yun, Guangzhou, China | Bathroom/washbasin |
| 264 | A423-3 | - | Unknown species | *Phialophora europaea*（C78%, I95%) | Bai Yun, Guangzhou, China | Kitchen/water tank |
| 265 | A223-1 | - | Unknown species | *Knufia perforans* (C99%, I86%) | Hai Zhu, Guangzhou, China | Kitchen/water tank |
| 266 | A345-1 | - | Unknown species | *Herpotrichiellaceae sp.* (C78%, I99%) | Hai Zhu, Guangzhou, China | Kitchen/water tank |
| 267 | A350-5 | - | Unknown species | *Phialophora europaea*（C79%, I95%) | Hai Zhu, Guangzhou, China | Bathroom/washbasin |
| 268 | A351-2 | - | Unknown species | *Phialophora europaea*（C79%, I95%) | Hai Zhu, Guangzhou, China | Bathroom/washbasin |
| 269 | A351-3 | - | Unknown species | *Phialophora europaea*（C79%, I95%) | Hai Zhu, Guangzhou, China | Bathroom/washbasin |
| 270 | A353-3 | - | Unknown species | *Phialophora europaea*（C79%, I95%) | Hai Zhu, Guangzhou, China | Bathroom/wall |
| 271 | A174-4 | - | Unknown species | *Knufia perforans* (C99%, I86%) | Hai Zhu, Guangzhou, China | Bathroom/wall |
| 272 | A175-3 | - | Unknown species | *Phialophora europaea*（C66%, I98%) | Hai Zhu, Guangzhou, China | Bathroom/wall |
| 273 | A177-1 | - | Unknown species | *Exophiala lecanii-corni* (C63%, I94%) | Hai Zhu, Guangzhou, China | Kitchen/water tank |
| 274 | A180-2 | - | Unknown species | *Phialophora europaea*（C79%, I95%) | Hai Zhu, Guangzhou, China | Bathroom/wall |
| 275 | A464-1 | - | Unknown species | *Phialophora europaea（*C80%, I94%) | Hai Zhu, Guangzhou, China | Bathroom/washbasin |
| 276 | A466-1 | - | Unknown species | *Phialophora europaea*（C79%, I95%) | Hai Zhu, Guangzhou, China | Bathroom/wall |
| 277 | A466-2 |  | Unknown species | *Phialophora europaea*（C79%, I95%) | Hai Zhu, Guangzhou, China | Bathroom/wall |
| 278 | A5-3 | - | Unknown species | *Knufia perforans* (C100%, I90%) | Hai Zhu, Guangzhou, China | Kitchen/water tank |
| 279 | A18-3 | - | Unknown species | *Phialophora europaea*（C80%, I95%) | Hai Zhu, Guangzhou, China | Bathroom/wall |
| 280 | A19-3-1 | - | Unknown species | *Phialophora europaea*（C80%, I95%) | Hai Zhu, Guangzhou, China | Bathroom/wall |
| 281 | A30-1 | - | Unknown species | *Phialophora europaea*（C80%, I95%) | Hai Zhu, Guangzhou, China | Bathroom/washbasin |
| 282 | A31-2 | - | Unknown species | *Phialophora europaea*（C81%, I95%) | Hai Zhu, Guangzhou, China | Bathroom/washbasin |
| 283 | A31-3 | - | Unknown species | *Knufia perforans* (C99%, I90%) | Hai Zhu, Guangzhou, China | Bathroom/washbasin |
| 284 | A33-1 | - | Unknown species | *Phialophora europaea*（C80%, I95%) | Hai Zhu, Guangzhou, China | Wash machine/rubber seal |
| 285 | A83-1 | - | Unknown species | *Exophiala sp.* (C91%, I86%) | Hai Zhu, Guangzhou, China | Bathroom/toothbrush cup |
| 286 | A83-4 | - | Unknown species | *Exophiala sp.* (C98%, I90%) | Hai Zhu, Guangzhou, China | Refrigerator/rubber seal |
| 287 | A88-1 | - | Unknown species | *Phialophora europaea*（C79%, I95%) | Hai Zhu, Guangzhou, China | Kitchen/water tank |
| 288 | A303-3 | - | Unknown species | *Phialophora europaea*（C80%, I94%) | Hai Zhu, Guangzhou, China | Kitchen/water tank |
| 289 | A303-4 | - | Unknown species | *Phialophora europaea*（C79%, I95%) | Hai Zhu, Guangzhou, China | Kitchen/water tank |
| 290 | A62-1 | - | Unknown species | *Phialophora europaea*（C80%, I95%) | Hai Zhu, Guangzhou, China | Kitchen/water tank |
| 291 | A298-1 | - | Unknown species | *Phialophora europaea*（C80%, I95%) | Hai Zhu, Guangzhou, China | Kitchen/water tank |
| 292 | Y10-1 | - | Unknown species | *Phialophora europaea*（C95%, I94%) | Hai Zhu, Guangzhou, China | Bathroom/washbasin |
| 293 | Y12-2 | - | Unknown species | *Phialophora europaea*（C95%, I94%) | Hai Zhu, Guangzhou, China | Bathroom/washbasin |
| 294 | A313-3 | - | Unknown species | *Phialophora europaea*（C78%, I95%) | Hai Zhu, Guangzhou, China | Bathroom/washbasin |
| 295 | A313-4 | - | Unknown species | *Herpotrichiellaceae sp.* (C78%, I99%) | Hai Zhu, Guangzhou, China | Bathroom/washbasin |
| 296 | A317-1 | - | Unknown species | *Phialophora europaea*（C80%, I95%) | Hai Zhu, Guangzhou, China | Kitchen/water tank |
| 297 | A317-2 | - | Unknown species | *Phialophora europaea*（C79%, I95%) | Hai Zhu, Guangzhou, China | Kitchen/water tank |
| 298 | A251-1-1 | - | Unknown species | *Phialophora europaea*（C79%, I95%) | Li Wan, Guangzhou, China | Bathroom/wall |
| 299 | A359-3 | - | Unknown species | *Phialophora europaea*（C81%, I95%) | Li Wan, Guangzhou, China | Kitchen/Chopping board |
| 300 | A359-5 | - | Unknown species | *Knufia perforans* (C99%, I90%) | Li Wan, Guangzhou, China | Kitchen/Chopping board |
| 301 | A359-6 | - | Unknown species | *Phialophora europaea*（C80%, I95%) | Li Wan, Guangzhou, China | Kitchen/Chopping board |
| 302 | A363-1 | - | Unknown species | Chaetothyriales sp. (C99%, I93%) | Li Wan, Guangzhou, China | Kitchen/water tank |
| 303 | A366-2 | - | Unknown species | *Herpotrichiellaceae sp.* (C79%, I99%) | Li Wan, Guangzhou, China | Bathroom/toothbrush cup |
| 304 | A373-4 | - | Unknown species | *Cyphellophora eucalypti* (C92%, I96%) | Li Wan, Guangzhou, China | Bathroom/washbasin |
| 305 | A387-2 | - | Unknown species | *Phialophora europaea*（C80%, I95%) | Li Wan, Guangzhou, China | Bathroom/toothbrush cup |
| 306 | A399-1 | - | Unknown species | *Exophiala lecanii-corni* (C64%, I94%) | Li Wan, Guangzhou, China | Kitchen/water tank |
| 307 | A117-3 | - | Unknown species | *Knufia perforans* (C98%, I90%) | Tian He, Guangzhou, China | Kitchen/water tank |
| 308 | A119-1 | - | Unknown species | *Knufia perforans* (C99%, I90%) | Tian He, Guangzhou, China | Kitchen/Chopping board |
| 309 | A128-3 | - | Unknown species | *Phialophora europaea*（C79%, I95%) | Tian He, Guangzhou, China | Bathroom/wall |
| 310 | A189-2 | - | Unknown species | *Phialaphora sp.* (C81%, I100%) | Yue Xiu,Guangzhou, China | Bathroom/wall |
| 311 | A198-1 | - | Unknown species | *Chaetothyriales sp.* (C99%, I91%) | Yue Xiu,Guangzhou, China | Refrigerator/rubber seal |
| 312 | A198-3 | - | Unknown species | *Chaetothyriales sp.* (C99%, I92%) | Yue Xiu,Guangzhou, China | Refrigerator/rubber seal |
| 313 | A199-1 | - | Unknown species | *Chaetothyriales sp*. (C100%, I99%) | Li Wan,Guangzhou, China | Kitchen/Chopping board |

1. The nucleotides differences showed as % for aligned nucleotides.
2. The nucleotides similarity showed for coverage (C%) and identity (I%).
